# Hypertriglyceridemia and hyperuricemia: a retrospective study of urban residents

**DOI:** 10.1186/s12944-019-1031-6

**Published:** 2019-04-01

**Authors:** Yan-long Hou, Xiao-lan Yang, Chun-xia Wang, Li-xia Zhi, Mei-juan Yang, Chong-ge You

**Affiliations:** 1 Laboratory Medicine Center, Lanzhou University Second Hospital, No. 82 Cuiyingmen Lanzhou, Lanzhou, 730030 Gansu China; 2 Department of Clinical Laboratory, The First People’s Hospital of Baiyin, Baiyin, 730900 Gansu China; 3 Department of Clinical Laboratory, The First People’s Hospital of Lanzhou city, Lanzhou, 730030 Gansu China; 4 Department of Clinical Laboratory, The Second People’s Hospital of Lanzhou city, Lanzhou, 730030 Gansu China

**Keywords:** Hyperuricemia; triglyceride, Uric acid, Metabolism

## Abstract

**Background:**

The aim of this study was to determine the association between hypertriglyceridemia and hyperuricemia (HUA).

**Methods:**

The study was conducted in 3884 subjects who had not received medication enrolled as a baseline. Each participant received at least three annual health check-ups between 2011 and 2017. The risk of hyperuricemia was assessed in four Quartiles (Q1 to Q4) according to TG levels using multivariate-adjusted logistic regression models.

**Results:**

The total incidence rate of HUA was 62.3/1000 person-years. In the univariate analysis, the risk of hyperuricemia in people with hypertriglyceridemia was 2.353 times that of normal triglycerides, with a 95% confidence interval of (2.011, 2.754), and the risk of hyperuricemia in men was 1.86 times of female, and the 95% confidence interval is (1.634, 2.177). After adjusting the potential confounders, the relative risk RR of TG at Q2 Q3 Q4 was 1.445 (95%CI:1.114, 1.901), 2.075 (1.611, 2.674), 2.972 (2.322, 3.804).

**Conclusions:**

TG is an independent risk factor for hyperuricemia. As the level of TG increases, the risk of HUA increases.

## Background

With the improvement of people’s living standards, the prevalence of metabolic diseases is also increasing. Hyperuricemia and hypertriglyceridemia are metabolic diseases. The prevalence of hyperuricemia in the world has been increasing over the past few decades [1]. Serum uric acid (SUA) is mainly produced by purines metabolism, and it has been proven to play emerging roles in human disease including metabolic syndrome and gout [2–4]. When the concentration of urate in the extracellular fluid is too high, urate crystals are formed, which leads to gout and seriously affects the quality of life. The level of serum uric acid is determined by production and the net balance of reabsorption or secretion by the kidney and intestine [5]. There are many risk factors for hyperuricemia. Some studies have shown that obesity, hypertension, total cholesterol, serum bisphenol A (Bisphenol A) and alcohol intake may increase the risk of HUA [6]. Intake of vitamin C and dairy products may lower serum uric acid levels [7–9]. Hypertriglyceridemia is a disorder of lipoprotein metabolism, which refers to serum triglyceride > 1.70 mmol / L, and it is a risk factor for cardiovascular disease and diabetes [10]. Japanese scholars have studied middle-aged men and pointed out that high triglycerides are risk factors for hyperuricemia [7]. Hypertriglyceridemia and hyperuricemia are also associated with cardiovascular disease, hypertension, obesity and insulin resistance [11–13]. This study used a complete physical examination data to establish a retrospective study to estimate the relationship between hypertriglyceridemia and hyperuricemia.

## Methods

### Study population

In this study, the data were selected from the medical examination population in the hospital in 2011. The criteria for personnel selection was more than two follow-ups since 2011. The first physical examination data of subjects was complete, and all subject did not receive medication, with a total of 3884 people serving as study, including 2060 males (age: 46.40 ± 11.29) and 1824 females (age: 42.24 ± 10.70), a total of 47 people was failed to follow up finally, and the rate of loss of follow-up was 1.2%. The first physical examination data was used as the baseline, and the study was divided into the normal group (*n* = 1686) and the elevated triglyceride group (*n* = 2198) according to the initial triglyceride level. Meanwhile, the study was divided into the HUA group (*n* = 720) and non-HUA group (*n* = 3164) according to whether the outcome occurred or not. The study was approved by the Ethics Committee of the Lanzhou University Second Hospital.

### Definition

HUA criteria: serum uric acid (SUA) male ≥420 μmol / L, female ≥360 μmol / L. Abnormal criteria for other indicators: BMI ≥ 25 kg/m^2^; blood urea nitrogen (BUN) > 7.1 mmol / L; serum creatinine (CR) men > 106 μmol / L, women > 97 μmol / L; blood glucose (GLU) > 7.0 mmol / L; triglycerides (TG) > 1.7 mmol/L; total cholesterol (CHOL) > 5.72 mmol/L; high-density lipoprotein (HDL) < 0.90 mmol/L; low-density lipoprotein (LDL) > 3.12 mmol/L.

### Measurement

All items were measured with an automatic biochemical analyzer (HITACHI 7600, JAPAN).

### Statistical methods

Data processing was performed using EXCEL and SPSS 22.0. The baseline characteristics and outcomes were statistically described as mean ± standard deviation (SD). The continuous variables were tested by t-test and the categorical variables were analyzed by *χ*^2^ test. The relationship between TG and HUA was analyzed by Cox regression model. A *P* value less than 0.05 was considered statistically significant.

## Results

1. Baseline characteristics of the study population are shown in Table 1. The baseline characteristics of the TG normal group and the TG elevated group were significantly different (*p* < 0.001).
2. Ending characteristics of the study population description are shown in Table 2. Among males, BMI, serum creatinine, blood uric acid and triglyceride in HUA group were higher than that in the non-HUA group; in women, HUA group age, serum creatinine, blood uric acid, blood sugar, triglyceride and total cholesterol were higher than non-HUA groups, and all had significant differences (*p* < 0.001).
3. The incidence of HUA with different gender with age is shown in Table 3. The incidence of women increases with age, and the change in men is not significant. The increase in the incidence of women is later than that of men.
4. Single factor analysis of hyperuricemia is shown in Table 4. The risk of hyperuricemia with hypertriglyceridemia is 2.353 times that of normal triglycerides, 95% confidence interval is (2.011, 2.754), People with hypercholesterolemia are 1.385 times more likely to develop hyperuricemia than normal people, and the risk of hyperuricemia in men is 1.886 times that of women, 95% confidence interval is (1.634, 2.177).
5. The incidence of HUA in different TG populations is shown in Table 5. The TG levels were divided into Q1, Q2, Q3, and Q4 from low to high. With the TG level increased, the incidence density also increased.
6. TG and HUA incidence correlation Cox regression analysis results are shown in Table 6.

**Table 1:** Baseline characteristics of the study population

Variables	TG normal	TG increase	*P* Valve
AGE(mean ± SD, years)	42.64 ± 11.93	45.84 ± 10.46	< 0.001
BMI(kg/m^2^)	22.47 ± 2.77	23.52 ± 2.17	< 0.001
BUN(mmol/L)	4.69 ± 1.21	4.83 ± 1.19	< 0.001
CREA(μmol/L)	64.65 ± 14.76	69.17 ± 15.01	< 0.001
UA(μmol/L)	274.17 ± 71.87	315.86 ± 73.76	< 0.001
GLU(mmol/L)	5.31 ± 1.06	5.65 ± 1.34	< 0.001
TG(mmol/L)	1.04 ± 0.35	2.27 ± 1.36	< 0.001
CHOL(mmol/L)	4.41 ± 0.78	4.88 ± 0.88	< 0.001
HDL(mmol/L)	1.47 ± 0.32	1.32 ± 0.28	< 0.001
LDL(mmol/L)	2.12 ± 0.62	2.37 ± 0.68	< 0.001

**Table 2:** Outcome characteristics of the study population

Variables	Male			Female		
	HUA(*n* = 490)	NORMAL(*n* = 1570)	*p*	HUA(*n* = 230)	NORMAL(*n* = 1594)	*p*
AGE(mean ± SD, years)	45.55 ± 11.16	46.66 ± 11.32	0.056	45.78 ± 12.65	41.73 ± 10.29	< 0.001
BMI(kg/m^2^)	24.46 ± 2.01	23.11 ± 2.11	< 0.001	24.17 ± 2.36	24.35 ± 1.95	0.211
BUN (mmol/L)	5.09 ± 1.15	5.06 ± 1.16	0.711	4.58 ± 1.11	4.39 ± 1.16	0.017
CREA(μmol/L)	79.33 ± 11.97	76.34 ± 11.21	< 0.001	62.04 ± 11.11	57.86 ± 9.90	< 0.001
UA(μmol/L)	366.29 ± 39.40	320.88 ± 52.32	< 0.001	324.53 ± 59.64	245.16 ± 49.99	< 0.001
GLU (mmol/L)	5.57 ± 1.35	5.75 ± 1.56	0.028	5.61 ± 1.08	5.21 ± 0.71	< 0.001
TG (mmol/L)	2.16 ± 1.33	1.90 ± 1.44	< 0.001	2.01 ± 1.31	1.31 ± 0.76	< 0.001
CHOL (mmol/L)	4.72 ± 0.84	4.63 ± 0.8	0.036	4.92 ± 0.89	4.57 ± 0.86	< 0.001
HDL (mmol/L)	1.21 ± 0.24	1.24 ± 0.25	0.030	1.39 ± 0.29	1.44 ± 0.30	0.008
LDL (mmol/L)	2.26 ± 0.63	2.29 ± 0.63	0.416	2.36 ± 0.68	2.11 ± 0.65	< 0.001

**Table 3:** Gender incidence with age

YEAR	CNP	CI	PR	ID(/1000PR)
2011	M 101	4.90	2060	49.0
	F 82	4.39	1864	43.9
2012	M 289	14.03	4120	70.1
	F 102	5.59	3648	27.9
2013	M 481	23.35	6180	77.8
	F 182	9.98	5472	33.3
2014	M 486	23.59	6318	76.9
	F 229	12.55	5588	40.9
2015	M 489	23.73	6354	76.9
	F 230	12.61	5606	41.0
2016	M 490	23.78	6366	76.9
	F 230	12.61	5611	40.9

Abbreviations: *M* male, *F* female, *CNP* Cumulative number of people, *CI* cumulative incidence, *PR* person year, *ID* incidence density

**Table 4:** Single factor analysis of hyperuricemia

Variables	TYPE	HUA		*p*	RR	95%CI
		YES	NO			
HIGH-TG	YES	543	1655	< 0.001	2.353	2.011–2.754
	NO	177	1509			
HIGH-CHOL	YES	104	318	0.001	1.385	1.018–1.675
	NO	616	2846			
HIGH-BUN	YES	29	114	0.584	0.911	0.654–1.269
	NO	691	3050			
HIGH-CR	YES	7	18	0.205	0.66	0.351–1.242
	NO	713	3146			
HIGH-GLU	YES	38	174	0.856	1.036	0.771–1.393
	NO	682	2990			
LOW-HDL	YES	32	112	0.274	0.828	0.605–1.132
	NO	688	3052			
HIGH-LDL	YES	70	283	0.518	1.077	0.864–1.344
	NO	650	2881			
GENDER	YES	490	1570	< 0.001	1.886	1.634–2.177
	NO	230	1594			

*CI* confidence interval

**Table 5:** HUA status of different TG levels

TG level(mmol/L)	Baseline number	HUA IN	PERSON-YEAR	ID(/1000PR)
Q1(<=0.97)	988	91	2694	33.7
Q2(0.98–1.39)	965	136	2944	46.2
Q3(1.40–2.04)	969	202	3091	65.3
Q4(> 2.04)	962	291	2823	103.1
Sum	3884	720	11,552	62.3

Quartiles are based on baseline triglyceride levels: Q1(quartile 1),< 0.97 mmol/L; Q2(quartile 2), 0.98–1.39 mmol/L;Q3(quartile 3), 1.40–2.04 mmol/L; Q4(quartile 4),> 2.04 mmol/L

**Table 6:** Analysis of associations between baseline triglyceride levels and incident HUA (OR: 95% CI)

Model	Q1(<=0.97)	Q2(0.98–1.39)	Q3(1.40–2.04)	Q4(> 2.04)
Model 1	1	1.529(1.172, 1.996)	2.301(1.792, 2.953)	3.461(2.729, 4.390)
Model 2	1	1.455(1.114, 1.900)	2.074(1.610, 2.673)	2.926(2.286, 3.746)
Model 3	1	1.455(1.114, 1.901)	2.075(1.611, 2.674)	2.972(2.322, 3.804)
*p*	< 0.001	< 0.01	< 0.001	< 0.001

Model 1: Adjusted for age; Model 2: Adjusted for age, BMI, gender, total cholesterol, high-density lipoprotein, low-density lipoprotein; Model 3: Adjusted for age, BMI, gender, total cholesterol, high-density lipoprotein, low-density lipoprotein, blood Urea nitrogen, serum creatinine, blood sugar

## Discussion

The characteristics of the outcomes in this study (Table 2) showed that the male and female from the HUA group and the non-HUA group had different degrees of difference in various indicators. For males, BMI, serum creatinine, blood uric acid, and triglyceride levels were higher for those in the HUA group than for those in the non-HUA group; among women, age, serum creatinine, serum uric acid, blood glucose, triglyceride, and total cholesterol levels were higher for those in the HUA group than for those in the non-HUA group. The possible reason for the gender difference is that the number of populations is insufficient. Another possible cause is estrogen. Some studies have shown that women before the menopause presented uric acid levels of around 60 μmol / L, which is lower than men’s. High levels of estrogen may promote excretion of uric acid, more effective removal of urate, and an increase in uric acid level after menopause [14]. Similarly, uric acid levels have decreased in men treated with estrogen, and the specific mechanism of the action of estrogen remains to be further studied [15, 16]. Body mass index (BMI) was associated with the prevalence of hyperuricemia, especially in the middle-aged, urban Chinese men [17]. In our study, the population with hyperuricemia had higher BMI for men. However, there is no significant difference in BMI among women. It might be related to men’s weight gain in adulthood and alcohol consumption.

Table 3 shows that there is a gender difference in the occurrence of hyperuricemia. The male incidence rate is higher than females’, which may be related to factors such as diet (there are more males than females who smoke) and physical condition (height and weight). Moreover, the incidence of HUA (18.5%) in this study was higher than that in rural areas of Northeast China (10.9%) and mainland China (13.3, 19.4% in men and 7.9% in women), which may be related to population structure, economic development and eating habits since rapid economic growth affects previous lifestyles [18, 19]. Hyperuricemia was more common in urban populations than in rural populations and the incidence was higher in inland areas than in coastal areas [20].

The incidence density of hyperuricemia increases significantly with higher triglyceride levels, suggesting that lowering triglyceride levels may reduce the risk of hyperuricemia. This result is consistent with the results of Yuan Zhang et al. [21]. Although the specific mechanism of the increase of TG level and hyperuricemia has not been elucidated. Some scholars have suggested that it may be due to the disorder of free fatty acids metabolism caused by triglyceride, as the increase of triglyceride leads to increased free fatty acid production. Accelerating the decomposition of adenosine triphosphate, which leads to an increase in uric acid, the end product of purine metabolism [22]. Some studies have shown that the synthesis of fatty acids in the liver is linked to the synthesis of the de novo purine and the accelerated production of urea [23]. The effects of triglyceride metabolic disorder on hyperuricemia remain to be further elucidated. Numerous factors may affect serum triglyceride levels, include high blood pressure, body mass index (BMI), age, gender, obesity, diet, lifestyle, and many other potential factors [24–26].

In this study, blood glucose, blood urea nitrogen, serum creatinine, high-density lipoprotein, and low-density lipoprotein were not significantly associated with HUA (*p* > 0.05). Triglycerides, total cholesterol, and gender were associated with the occurrence of HUA (*p* < 0.05). The incidence density and OR values were 103.1/1000 person-years and 2.972 (model 3), respectively, which were higher than these of TG levels at Q1, Q2, and Q3.

The results of this study showed that after adjusting for confounding factors, the occurrence of HUA was significantly affected when the TG level is significantly higher than the normal range. Therefore, strengthening the monitoring of serum TG levels can be used as a basis for preventing HUA.

The innovation of this study is that it uses more than 3 years of study data, which are more valuable than the case-control study results. In addition, this study compares the initial baseline and outcome characteristics of the population to enhance the persuasiveness of the conclusion.

The limitation of this study is the missing data and the physical examination data are not enough to fulfill the requirement of completeness. There are many important factors affecting hyperuricemia, but the diagnosis is only based on a single examination data, so there will be bias. In addition, the data collected is not complete, there are no data on smoking, drinking, and other socio-economic factors, and thus the description of the population’s characteristics is lacking.

## Conclusion

TG is an independent risk factor for HUA. As the level of TG increases, the risk of HUA increases.

## Abbreviations

BMI: Body mass index
BUN: Blood urea nitrogen
CHOL: Total cholesterol
GLU: Blood glucose
HDL: High-density lipoprotein
LDL: Low-density lipoprotein
SCR: Serum creatinine
SUA: Serum uric-acid
TG: Triglycerides
